# Simultaneous Determination of Bufalin and Its Nine Metabolites in Rat Plasma for Characterization of Metabolic Profiles and Pharmacokinetic Study by LC–MS/MS

**DOI:** 10.3390/molecules24091662

**Published:** 2019-04-28

**Authors:** Wenlong Wei, Yang Yu, Xia Wang, Linhui Yang, Hang Zhang, Hongjian Ji, Zhenwei Li, Jinjun Hou, Wanying Wu, Dean Guo

**Affiliations:** Shanghai Research Center for Modernization of Traditional Chinese Medicine, National Engineering Laboratory for TCM Standardization Technology, Shanghai Institute of Materia Medica, Chinese Academy of Science, Shanghai 201203, China; 13521032532@163.com (W.W.); 201728012342031@simm.ac.cn (Y.Y.); xiawang7788@163.com (X.W.); ylhwssy@126.com (L.Y.); lisa123456789love@163.com (H.Z.); hongjianji2006@163.com (H.J.); 201628012342031@simm.ac.cn (Z.L.); jinjun_hou@simm.ac.cn (J.H.)

**Keywords:** bufalin, metabolite, quantification, pharmacokinetics

## Abstract

Characterization and determination of metabolites to monitor metabolic pathways play a paramount role in evaluating the efficacy and safety of medicines. However, the separation and quantification of metabolites are rather difficult due to their limited contents in vivo, especially in the case of Chinese medicine, due to its complexity. In this study, an effective and convenient method was developed to simultaneously quantify bufalin and its nine metabolites (semi-quantitation) in rat plasma after an oral administration of 10 mg/kg to rats. The prototype and metabolites that were identified were subsequently quantified using positive electrospray ionization in multiple reaction monitoring (MRM) mode with transitions of *m*/*z* 387.4→369.6 and 387.4→351.3 for bufalin, *m*/*z* 513.7→145.3 for IS, and 387.4→369.6, 419.2→365.2, and 403.2→349.2 for the main metabolites (3-epi-bufalin, dihydroxylated bufalin, and hydroxylated bufalin, respectively). The method was validated over the calibration curve range of 1.00–100 ng/mL with a limit of quantitation (LOQ) of 1 ng/mL for bufalin. No obvious matrix effect was observed, and the intra- and inter-day precisions, as well as accuracy, were all within the acceptable criteria in this method. Then, this method was successfully applied in metabolic profiling and a pharmacokinetic study of bufalin after an oral administration of 10 mg/kg to rats. The method of simultaneous determination of bufalin and its nine metabolites in rat plasma could be useful for pharmacokinetic–pharmacodynamic relationship research of bufalin, providing experimental evidence for explaining the occurrence of some adverse effects of *Venenum Bufonis* and its related preparations.

## 1. Introduction

*Venenum Bufonis* (VB), named *Chansu* in Chinese, a product of the secretions of *Bufo gargarizans* Cantor or *Bufo melanostictus* Schneider, is a well-known traditional Chinese medicine (TCM), which was widely used in Asia. It has been used for the treatment of ulcers and deep-rooted boils, swelling and pain in the throat, sunstroke and fainting, and acute filthy disease—abdominal pain or vomiting and diarrhea, for thousands of years in China [1]. In recent years, some studies revealed that VB could also be used as an anesthesia analgesic or cardiac stimulant, and had great potential to be developed as anti-tumor drugs [2,3,4]. Bufalin, one of the major active components in VB, is regarded as a vital marker compound for the quality control of VB, along with its related TCM preparations [5]. Arrays of studies have shown its powerful cytotoxic activities against various tumor cells, such as human leukemia cells, lung cancer cells, human hepatocellular carcinoma HepG2 cells, human malignant melanoma A375.S2 cells, etc. [6,7,8]. Meanwhile, bufalin possesses powerful cardiotoxicity and neurotoxicity, and it can result in convulsion, diarrhea, arrhythmia, and other symptoms [9,10].

The metabolism of a given drug and the formation of its metabolites may significantly influence the efficacy and toxicity of other drugs in vivo. Some studies regarding the metabolic profiles of bufalin have demonstrated that hydroxylation and dehydrogenation mediated by CYP3A metabolic enzymes were two major metabolic pathways of bufalin in liver microsomes from human and animal species [11,12,13]. Furthermore, the metabolites of bufalin exhibited a strong selective inhibition effect on the CYP3A4 metabolic enzyme, which could induce the function loss of CYP3A4 and result in severe drug–drug interactions [14]. Therefore, it is of pivotal importance to monitor the metabolic profiles of bufalin and its metabolites for the pharmacodynamic and safety evaluation. Although several studies have focused on the identification of metabolites and determination of bufalin [15,16,17], there has been no report on the simultaneous determination of bufalin and its metabolites for characterization of metabolic profiles, due to the difficulty in separation and quantification of low-content metabolites in vivo. 

In this study, an effective and convenient method was developed to simultaneously determine bufalin and its nine metabolites in rat plasma by LC–MS/MS. The method was composed of three steps: Firstly, raw data of plasma samples were collected on UPLC–QTOF/MS and subsequently processed by UNIFI for characterization of metabolites. Secondly, metabolites identified by high-resolution MS were analyzed by LC–MS/MS for better quantification after optimization of conditions to obtain the characteristic ion pairs of metabolites in multiple reaction monitoring (MRM) mode. Lastly, bufalin and its nine metabolites in rat plasma were determined simultaneously for characterization of metabolic profiles and pharmacokinetic study. 

## 2. Materials and Methods 

### 2.1. Chemicals and Reagents

**Figure 1:**
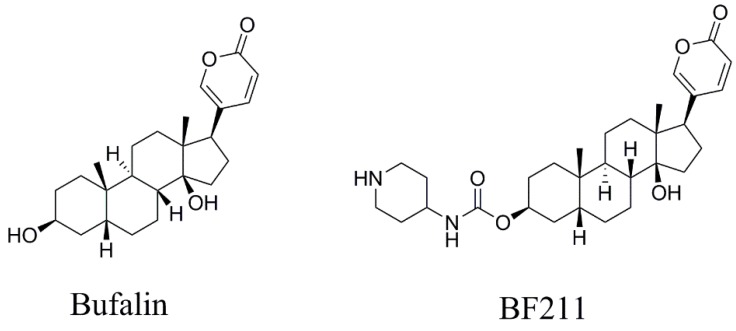
The chemical structures of bufalin and BF211.

Bufalin was isolated from *Venenum Bufonis*, and BF211 (internal standard, IS) was synthesized in our laboratory [18]. Their structures were identified by detailed high-resolution MS and NMR analysis. Bufalin and BF211 showed >98% purity, as determined by HPLC-UV (Figure 1). Leucine-enkephalin was purchased from Sigma–Aldrich (St. Louis, MO, USA). HPLC-grade acetonitrile (Merck, Darmstadt, Germany), formic acid (ROE Scientific INC., Waltham, MA, USA), and ultra-pure water, which was prepared by Millipore Alpha-Q water purification system (Millipore, Bedford, MA, USA), were used in the mobile phase.

### 2.2. UPLC–QTOF/MS Instruments and Conditions

Chromatographic separation was performed on a Waters ACQUITY I-Class UPLC^®^ system (Waters Corporation, Milford, MA, USA), equipped with a binary solvent manager, a sample manager, and a column manager. An ACQUITY UPLC^®^ HSS T3 column (1.8 μm, 2.1 × 100 mm) equipped with an online filter was used and eluted by a binary mobile phase composed of acetonitrile (B) and 0.1% formic acid (*v*/*v*; A) following the gradient elution program: 0–2 min: 5% B; 2–4 min: 5–17% B; 4–6 min: 17–28% B; 6–9 min: 28–28% B; 9–14 min: 28–35% B; 14–20 min: 35–39% B; 20–21 min: 39–95%; 21–25 min: 95–95%. The column temperature was set at 30 °C. The flow rate was 0.5 mL/min, and 10 µL of the test solution was injected.

High-resolution profile MS data were acquired on a Waters Xevo^®^ G2-S QTOF mass spectrometer (Waters, Manchester, UK) connected to the UPLC system via a Zpray™ ESI source. The mass range of *m*/*z* 150–800 was set for full-scan, and the collision energy ramps of 15–25 V and 35–45 V were set for low mass and high mass, respectively. Capillary voltages of 2 kV, cone voltage of 40 V, cone gas flow of 30 L/h, source temperature of 140 °C, and desolvation gas flow of 700 L/h at 500 °C were utilized. A solution of leucine enkephalin (1 µg/mL) was used as lock mass for data calibration. Data acquisition and processing were performed using MassLynx V4.1 software (Waters, Manchester, UK). UNIFI software (Waters, Milford, MA, USA), a powerful platform that could be used for data acquisition, data mining, library searching, and metabolites predicting, was mainly applied for prediction and identification of metabolites in this study. The parameters were listed as follows: MS ion intensity threshold, 100 counts; MS/MS ion intensity threshold, 30 counts; target match tolerance, 5 mDa; fragment match tolerance, 5 mDa; selected adduct of mass defect filter, [M + H]^+^; mass padding of mass defect filter, 15 Da; defect padding of mass defect filter, 40 mDa; mass tolerance of binary comparison, 2 mDa; relative intensity threshold, 30%. Reference mass of lock mass setting, 556.2766 *m*/*z*. The transformations, which were included in UNIFI, are listed in Table S1.

### 2.3. LC–MS/MS Instruments and Conditions

Chromatographic separation was performed on an Agilent UHPLC system (Agilent Technologies, Palo Alto, CA) equipped with a binary pump, a sample manager, and a column manager. An ACQUITY UPLC^®^ BEH C18 column (1.7 µm, 2.1 × 50 mm) was used and eluted by a binary mobile phase composed of acetonitrile (B) and 0.05% formic acid (*v*/*v*; A) following the gradient elution program (0–9.5 min: 22-35% B; 9.5–9.9 min: 35–90% B; 9.9–10 min: 90–22% B; 10–11 min: 22–22% B). The column temperature was set at 40 °C. The flow rate was 0.4 mL/min, and 10 µL of the test solution was injected.

An Applied Biosystems 4000 QTRAP^®^ LC–MS/MS system (Toronto, Canada), including a hybrid triple quadrupole/LIT (linear ion trap) mass spectrometer, was equipped with a Turbo V™ ion in the positive mode for data collection. The shared mass spectrometry parameters were 35 psi curtain gas, 50 psi nebulizer gas, 50 psi heater gas, medium collision gas, 500 °C ion spray temperature, and 5500 V ion spray voltage. The instrument control, data acquisition, and original data processing were performed using AB Sciex Analyst 1.6.3 software (Framingham, MA, USA) [19,20].

### 2.4. Preparation of Calibration Standards and Quality Control (QC) Samples

Bufalin was accurately weighed and dissolved in 50% methanol-water (1:1, *v*/*v*) to obtain the stock solution with a concentration of 1 mg/mL, which was stepwise diluted with 50% methanol-water to yield working standard solutions at concentration levels of 20, 40, 100, 200, 400, 1000, and 2000 ng/mL. These standard solutions were all kept at 4 °C before usage. The stock solution of IS with a concentration of 1 mg/mL was prepared in the same way and diluted to 50 ng/mL. Each working standard solution (5 µL), blank rat plasma (95 µL), and IS working solution (10 µL) was added into a 1.5 mL centrifuge tube and mixed with a Vortex-2 machine for 3 min respectively, followed by an addition of 1 mL ethyl acetate for extraction and centrifugation at 3500 rpm for 10 min. Then, 900 µL of supernatant was transferred to a 2 mL centrifuge tube, and evaporated to dryness by N_2_ blowing. The residual mixture was reconstituted with 20% acetonitrile-water (100 µL) with vortex and centrifugation, and the supernatant was transferred to sample vials for analysis of calibration curves. The final calibration standards were 1, 2, 5, 10, 20, 50, and 100 ng/mL for bufalin. The lower limit of quantification (LLOQ) was 1 ng/mL, and the concentrations of QC samples at low, medium, and high levels were 3, 15, and 80 ng/mL respectively.

### 2.5. Sample Preparation

The 10 µL IS working solution and 100 µL plasma samples were added to a 1.5 mL centrifuge tube, followed by vortex (3 min) and centrifugation (3500 rpm, 10 min), and 1 mL ethyl acetate was added for extraction. Then, 900 µL supernatant was acquired and evaporated by N_2_ blowing, and the residue was reconstituted with 100 µL of 20% acetonitrile-water.

### 2.6. LC–MS/MS Method Validation

To ensure the acceptability of the performance and the reliability of the analytical results by the developed LC–MS/MS method, a full method validation, including specificity, accuracy, precision, recovery, matrix effect, and stability, were performed according to the guidelines set by the FDA.

#### 2.6.1. Selectivity

Selectivity of bufalin and IS was proved using 6 individual sources of blank plasma. The peak area at the retention time of the analyte in the blank plasma should be less than 20% of that of LLOQ in the plasma spiked with bufalin, while IS should be less than 5%.

#### 2.6.2. Accuracy and Precision

The intra- and inter-day precisions were determined by analyzing QC samples at LLOQ, low, medium, and high concentration levels on the same day and on three consecutive days, respectively (*n* = 6). The accuracy was calculated as the relative error (RE), which should be within ±15%. The intra- and inter-batch precisions were calculated as the coefficient of variation (CV) or relative standard deviation (RSD), which was required to not exceed 15% at low, medium, and high levels. Meanwhile, the RE and RSD of LLOQ should not exceed 20%.

#### 2.6.3. Recovery and Matrix Effect

Three QC concentration levels (low, medium, and high) were applied for evaluation of recovery and matrix effect. Recoveries of bufalin and IS were assessed by comparing the mean peak areas of the regular QC samples to that of post liquid–liquid-extracted blank plasma, spiked with the corresponding analyte in six replicates. The matrix factor was calculated as the ratio of the peak area in the presence of matrix to the mean peak area in the absence of matrix, and the IS normalized matrix factor was calculated by dividing the matrix factor of the bufalin by the matrix factor of IS.

#### 2.6.4. Stability

The stability of bufalin in rat plasma was evaluated by analyzing QC samples at low and high concentrations in six replicates under different storage conditions. The short-term stability was assessed by analyzing the QC samples that were kept for 4 h at ambient temperature. The autosampler stability was conducted by reanalyzing QC samples that were stored in the autosampler at 5 °C condition for 24 h. Freeze–thaw stability (−20 °C) was assessed after three freeze and thaw cycles, with at least 12 h between each cycle. Moreover, the low and high concentration samples were preserved at −20 °C (30 days) for evaluation of long-term stability. The analyte was considered stable when the bias (%) between stability samples and the theoretical values of low and high concentrations were within ±15%, according to the freshly prepared calibration curves.

### 2.7. Characterization of Metabolic Profiles and Pharmacokinetic Study in Rats

Male Wistar rats (200 ± 20 g) were purchased from SLAC Lab Animal Center (Shanghai, China). Rat studies were conducted according to protocols approved by the Review Committee of Animal Care and Use at the Shanghai Institute of Materia Medica (Shanghai, China). All rats used in the experiment were fed in rat cages in a unidirectional airflow room under controlled temperature (20–24 °C), a 12 h light/dark cycle, and relative humidity (40–70%). Food and water were offered ad libitum and rats were acclimated to the facilities and environment for 7 days before the experiments. Bufalin was dissolved in dimethyl sulfoxide (0.5% percent) and dispersed uniformly in sodium carboxymethylcellulose (0.5% g/mL). Three rats were orally administered a single dose of 10 mg/kg. The blood samples were collected into heparinized tubes at 0, 0.083, 0.25, 0.5, 0.75, 1, 2, 4, 6, 8, 10, and 24 h after oral administration and immediately placed in ice. After centrifuging, the supernatant fractions were transferred and stored at −20 °C until analysis. All experimental procedures were carried out in strict accordance with the NIH Guidelines for the Care and Use of Laboratory Animals, and all protocols were approved by the Institutional Animal Care and Use Committee of the Shanghai Institute of Materia Medica. The maximum plasma concentration (*C*_max_) was calculated on the basis of the measured values, and other pharmacokinetic parameters were analyzed by a statistical moment of Drug and Statistics (DAS) software (Version 2.0, Shanghai, China).

## 3. Results and Discussion

### 3.1. Identification of Metabolites of Bufalin

After oral administration of 10 mg/kg bufalin to rats, the plasma samples (*n* = 3) were obtained and mixed at 5 min, 15 min, 30 min, 45 min, 1 h, and 2 h points, in sequence. The raw data of plasma samples were acquired by UPLC–QTOF/MS, and then imported into UNIFI for analysis. The results showed that the main metabolic pathways of bufalin include isomerization and oxidation. The tentative characterization of metabolites of bufalin was listed in Table 1. The proposed metabolic pathways of bufalin are shown in Figure S1. Furthermore, the transformation of MRM settings of main metabolites from UPLC–QTOF/MS to LC–MS/MS is shown in Table 2.

### 3.2. LC–MS/MS Condition Optimization

**Figure 2:**
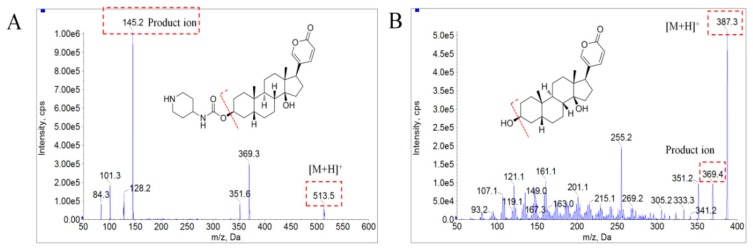
Mass spectrograms of product ions of bufalin (**B**) and BF211 (**A**) in positive mode.

The mass spectrometry conditions for bufalin and IS were optimized in positive modes with ESI source. The standard solutions were injected into the mass spectrometer, using a micro injection pump to select the precursor ions and product ions for the application in MRM mode. The protonated precursor ions [M + H]^+^ at *m*/*z* 387.4 and 513.7 in the full scan mass spectra were selected as precursor ions for bufalin and IS, due to their most abundant responses. In product ion scan mode, fragment ions at *m*/*z* 369.6 and 351.3, generated with dehydroxylation (3- and 14-position hydroxyl group), were chosen for bufalin, while a fragment ion at *m*/*z* 145.3, produced from the broken of 3-position ester bond group, was picked for IS (Figure 2). Meanwhile, the compound parameters, such as declustering potential (DP) and collision energy (CE), were optimized as 115 V and 41V for bufalin and 78 V and 26 V for IS to acquire higher sensitivity. The detailed optimization mass spectrometry conditions for bufalin and BF211 are shown in Table 3.

To obtain the optimal peak resolution and separation, as well as to increase the ionization intensity and shorten the elution time, the mobile phase additive, column temperature, and flow rate were optimized. Finally, 0.05% formic acid was selected and added to the aqueous phase, and column temperature and flow rate were optimized as 40 °C and 0.4 mL/min. More importantly, isomers of bufalin (3-epi-bufalin) and its metabolite-hydroxylated bufalin were fully separated under this optimized elution gradient.

### 3.3. Sample Preparation

The protein precipitation and liquid–liquid extraction (LLE) were compared to screen a suitable method for sample processing. LLE exhibited higher response intensity and less endogenous interference, which was consistent with previous studies [21]. Furthermore, three extracting solvents containing ethyl acetate, diethyl ether, ethyl acetate-isopropanol (9:1) were also compared, respectively. In consideration of response intensity and volatility together, LLE with ethyl acetate was selected as an effective and feasible approach for the extraction of bufalin and IS from rat plasma.

### 3.4. Method Validation

**Figure 3:**
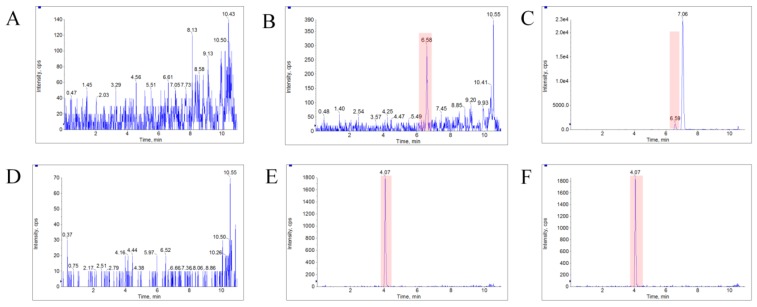
The typical MRM chromatograms of bufalin and IS. (**A**), (**B**), and (**C**) represent the bufalin in blank blood, blank plasma spiked with bufalin (LLOQ), and plasma sample from a rat with a single dose of 10 mg/kg bufalin, respectively. (**D**), (**E**), and (**F**) represent the IS, likewise.

The LC–MS/MS method was fully validated for selectivity, accuracy, precision, recovery, matrix effect, and stability. The typical MRM chromatograms of bufalin and IS were shown in Figure 3. The results showed that there was no obvious endogenous interference at the elution times of bufalin and IS, which were approximately 6.58 and 4.07 min, respectively, and no carryover effect was observed, either. The calibration curve used for semi-quantification showed good linearity over the concentration range of 1.00–100 ng/mL with a typical equation: ƒ = 0.131 × C + 0.0215 (*r* = 0.9990), in which ƒ represents the peak area ratio of bufalin to IS, and C represents the concentration of bufalin to IS. A 1/x^2^ weighted linear regression model was applied to ensure the best fitting for correlation between response and concentrations of bufalin. The intra- and inter-batch precision and accuracy data for the determination of bufalin at LLOQ and three QC levels are listed in Table 4, which showed that the method was accurate and precise for the determination of bufalin in rat plasma. The matrix effects of bufalin at low, medium, and high QC concentration levels were 96 ± 7%, 94 ± 4.7%, and 92 ± 11.9%, respectively, which indicated that the matrix effects for bufalin under this condition could be negligible. In addition, the extraction recoveries at low, medium, and high QC concentration levels were 88.2, 85.5, and 92.2 for bufalin, and 86.0 for IS, which indicated that the extracting method possessed high recoveries and consistency at three QC levels. It also supported the optimization of the extraction procedure for the application of sample analysis. The stability data of bufalin in rat plasma are listed in Table 5, and showed that no significant degradation occurred in rat plasma under different storage conditions.

### 3.5. Characterization of Metabolic Profiles and Pharmacokinetic Study in Rats

**Figure 4:**
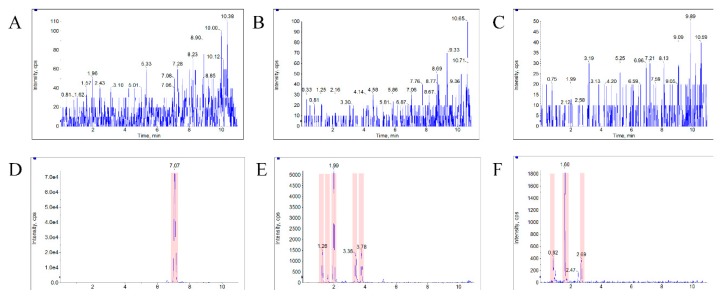
The typical MRM chromatograms of metabolites. (**A**), (**B**), and (**C**) represent the metabolites’ *m*/*z* 387.4–369.6, 403.25–349.22, and 419.24–365.21 in blank plasma, respectively. (**D**), (**E**), and (**F**) represent the three metabolites in a plasma sample from a rat with a single dose of 10 mg/kg bufalin.

**Figure 5:**
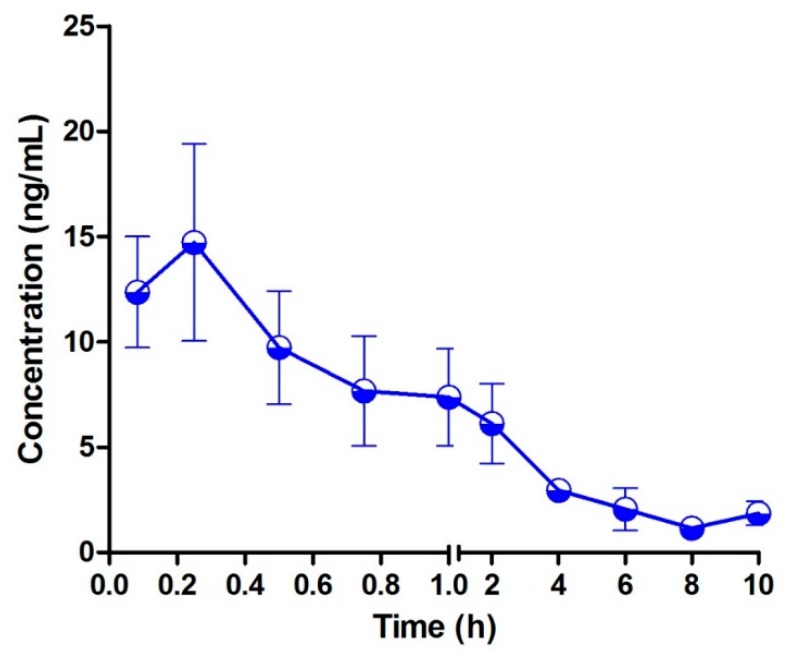
Mean plasma concentration–time profiles of bufalin in rats after oral administration of 10 mg/kg (*n* = 3).

**Figure 6:**
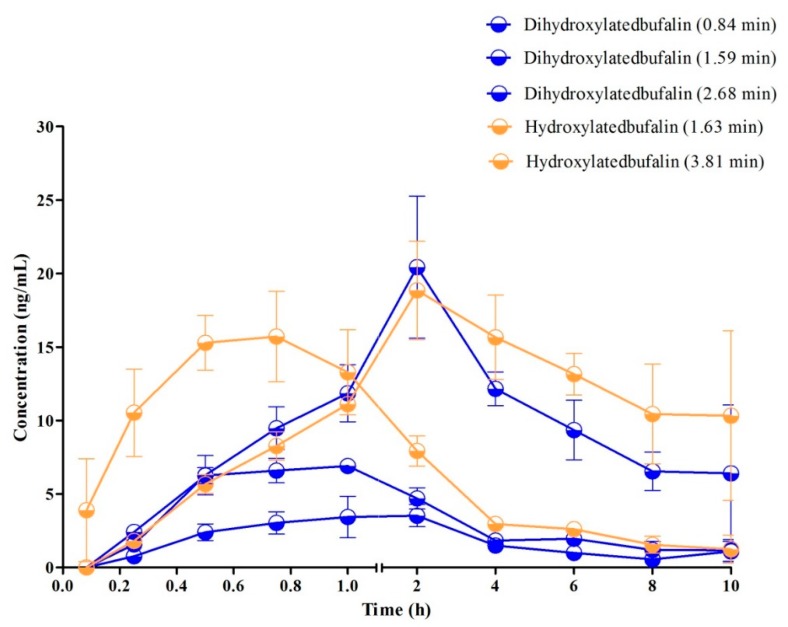
Mean plasma concentration–time profiles of metabolites (*C*_max_ was in the range of 2 to 25 ng/mL) in rats after oral administration of 10 mg/kg (*n* = 3).

**Figure 7:**
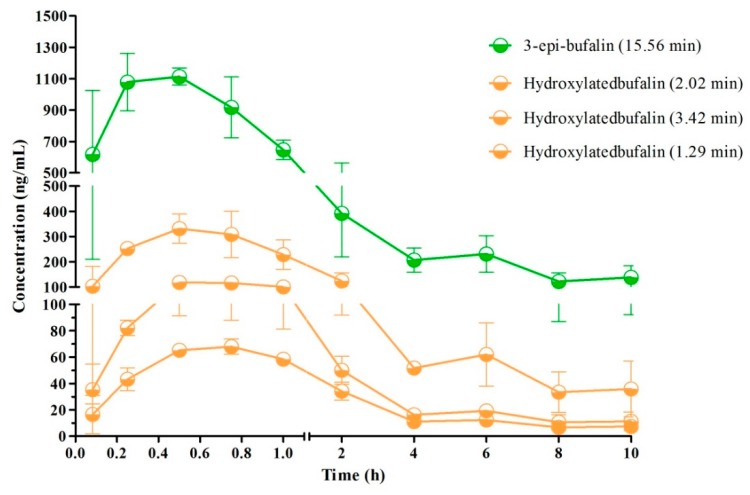
Mean plasma concentration–time profiles of metabolites (*C*_max_ was above 50 ng/mL) in rats after oral administration of 10 mg/kg (*n* = 3).

The typical MRM chromatograms of the metabolites are shown in Figure 4. These main metabolites (*m*/*z* 387.4–369.6, 403.2–349.2, 419.2–365.2) were not detected in blank blood, but could be clearly observed in plasma samples from a rat with a single dose of 10 mg/kg bufalin (Figure 4d–f). The bufalin and its nine metabolites were simultaneously determined, in order to study the pharmacokinetic behaviors of bufalin in rats after a single oral administration of 10 mg/kg. The semi-quantitation of metabolites was performed on the basis of the linear equation of bufalin. The area ratio of analyte to IS was substituted into the equation to calculate the concentration of metabolites, respectively (Tables S2 and S3). The concentration–time curves of bufalin and its main metabolites are displayed in Figure 5, Figure 6 and Figure 7, and the main pharmacokinetic parameters are exhibited in Table 6. From Figure 5 and Figure 7, it can be seen that bufalin was rapidly metabolized to 3-epi-bufalin at 5 min, and the *C*_max_ value (963.06 ± 284.76 ng/mL) of 3-epi-bufalin was well above the bufalin (14.72 ± 4.68 ng/mL). Moreover, three hydroxylated metabolites (retention time: 2.02, 3.42, and 1.29 min) also showed higher *C*_max_ values (299.56 ± 112.89, 107.36 ± 44.57, and 57.37 ± 13.41 ng/mL) than that of bufalin. In Figure 6, two hydroxylated metabolites and three dihydroxylated metabolites exhibited similar *C*_max_ values with bufalin in the range of 2–25 ng/mL. These results implied that the metabolites of bufalin (isomerization and hydroxylation) may largely result in the pharmacological and toxicological effects of bufalin in rats, due to their high exposure dose in the plasma, but the toxicity and efficacy of these metabolites need to be further verified. In addition, the absorption of bufalin was quick, and reached the *C*_max_ at 15 min. From Figure 6 and Figure 7, the results showed that the isomerization and hydroxylation mainly occurred within 30 min, and dihydroxylation occurred in the range of approximately 1–2 h. The hydroxylation and dihydroxylation mainly focused on the 1- and 5-positions [12,16], but the stereochemistry was difficult to distinguish among isomer metabolites. The main metabolites with high exposure doses should be isolated, and their active and toxic cell assays should also be performed to elucidate the pharmacological and toxicological effects of bufalin.

## 4. Conclusions

An effective and convenient LC–MS/MS method was developed and validated for the simultaneous determination of bufalin and its nine metabolites in rat plasma for the first time. Furthermore, this validated method was successfully applied in the pharmacokinetic study of bufalin after oral administration to rats. In the pharmacokinetic study, the absorption of bufalin was quick and the *C_max_* was reached within 0.25 h. The result was similar to that of previous studies. Furthermore, bufalin was rapidly transformed into 3-epi-bufalin, hydroxylated bufalin (RT = 2.02 min, and dihydroxylated bufalin (RT = 1.59 min), and the *C_max_* values were 963.06 ± 284.76, 299.56 ± 112.89, and 107.36 ± 44.57 ng/mL in rats. The main metabolites that possessed high blood concentration should be separated and isolated, and the toxicity and activity need to be further tested. The pharmacokinetic and metabolic profiles of bufalin could provide essential data for future research into its pharmacology, as well as its toxicology.

## Figures and Tables

**Table 1:** The tentative characterization of metabolites of bufalin in rat plasma.

NO.	Component Name	Pathway	Formula	M + H (*m*/*z*)	Retention Time (RT, min)	Fragments
1	3-epi-bufalin	Isomerization	C_24_H_34_O_4_	387.2527	15.56	369.24; 351.23; 333.22; 255.21
2	Unknown	Bufalin-O(cleavage) + H_2_	C_24_H_36_O_3_	373.2735	18.63	355.26; 337.25; 255.21
3	Unknown	Bufalin-O(cleavage) + H_2_	C_24_H_36_O_3_	373.2737	14.6	355.26; 337.25; 255.21
4	Unknown	Bufalin-O(cleavage) + H_2_	C_24_H_36_O_3_	373.2739	13.95	355.26; 337.25; 255.21
5	Unknown	Bufalin + 2 × H_2_	C_24_H_38_O_4_	391.2830	15.24	373.27; 355.26; 337.25
6	Unknown	Bufalin + 2 × H_2_	C_24_H_38_O_4_	391.2833	20.84	373.27; 355.26; 337.25
7	Hydroxylation-bufalin	Bufalin + O	C_24_H_34_O_5_	403.2473	9.65	385.24; 367.23; 349.22; 331.21; 253.19
8	Hydroxylation-bufalin	Bufalin + O	C_24_H_34_O_5_	403.2470	7.58	385.24; 367.23; 349.22; 331.21; 253.19
9	Hydroxylation-bufalin	Bufalin + O	C_24_H_34_O_5_	403.2471	6.75	385.24; 367.23; 349.22; 331.21; 253.19
10	Hydroxylation-bufalin	Bufalin + O	C_24_H_34_O_5_	403.2451	10.57	385.24; 367.23; 349.22; 331.21; 253.19
11	Unknown	Unknown	C_24_H_34_O_5_	403.2451	7.05	No found
12	Unknown	Unknown	C_24_H_34_O_5_	403.2492	9.87	No found
13	Dihydroxy-bufalin	Bufalin + 2 × O	C_24_H_34_O_6_	419.2424	7.01	401.24; 383.22; 365.21; 347.20
14	Unknown	bufalin-O + 2 × (-H_2_) + C_2_H_2_O	C_26_H_32_O_4_	409.2345	15.55	369.24; 351.23

**Table 2:** Transformation of multiple reaction monitoring (MRM) settings of main metabolites from UPLC–QTOF/MS to LC–MS/MS.

Metabolites	ESI Mode	QTOF/MS		MS/MS	
		Precursor ion (M + H)	Fragments	Precursor Ion	Product Ion
3-epi-bufalin	ES+	387.2527	369.24; 351.23; 333.22; 255.21	387.40	369.60
Dihydroxylated bufalin	ES+	419.2424	401.24; 383.22; 365.21; 347.20	419.24	365.21
Hydroxylated bufalin	ES+	403.2473	385.24; 367.23; 349.22; 331.21; 253.19	403.25	349.22
Hydroxylated bufalin	ES+	403.2470	385.24; 367.23; 349.22; 331.21; 253.19	403.25	349.22
Hydroxylated bufalin	ES+	403.2471	385.24; 367.23; 349.22; 331.21; 253.19	403.25	349.22
Hydroxylated bufalin	ES+	403.2451	385.24; 367.23; 349.22; 331.21; 253.19	403.25	349.22

**Table 3:** Optimized mass spectrometry conditions for bufalin and BF211.

Analyte	Q1 Mass (Da)	Q3 Mass (Da)	DP (Volts)	CE (Volts)
Bufalin	387.4	369.6	115	41
BF211	513.7	145.3	78	26

**Table 4:** Precision and accuracy for bufalin in rat plasma (*n* = 6).

Analyte.	Spiked Conc. (ng/mL)	Intra-Batch (n = 6)			Inter-Batch (*n* = 6 × 3)		
		Measured Conc. (mean ± SD, ng/mL)	Precision (RSD, %)	Accuracy (RE, %)	Measured Conc. (mean ± SD, ng/mL)	Precision (RSD, %)	Accuracy (RE, %)
**Bufalin**	1.00	1.08 ± 0.13	12.04	8.28	1.06 ± 0.11	10.28	6.09
	3.00	3.26 ± 0.17	5.10	8.61	3.26 ± 0.18	5.66	8.61
	15.00	16.42 ± 0.51	3.09	9.44	16.39 ± 0.69	4.19	9.30
	80.00	75.68 ± 3.66	4.83	−5.40	82.23 ± 5.95	7.23	2.79

**Table 5:** Stability of bufalin in rat plasma under various storage conditions (*n* = 6).

Analytes	Nominal Conc. (ng/mL)	Room Temperature Stability for 4 h		Autosampler Stability for 24 h		Freeze–Thaw Stability for Three Cycles at −80 °C		The Long-Term Stability at −80 °C for 25 Days	
		Precision (RSD, %)	Accuracy (RE, %)	Precision (RSD, %)	Accuracy (RE, %)	Precision (RSD, %)	Accuracy (RE, %)	Precision (RSD, %)	Accuracy (RE, %)
Bufalin	3.00	6.9	7.6	8.7	−5.1	6.0	4.0	6.2	6.0
	80.0	5.6	2.8	5.0	−11.4	−0.9	−4.0	4.8	−6.0

**Table 6:** Pharmacokinetics parameters of bufalin and its metabolites after a single oral administration of 10 mg/kg bufalin to rats (*n* = 3, mean ± SD).

Parameters	*C*_max_ (ng/mL)	*T*_max_ (h)	t_1/2_ (h)	AUC_0-t_ (ug/L∗h)	MRT_0-t_ (h)
Bufalin	14.722 ± 4.681	0.25 ± 0.00	5.70 ± 3.06	37.31 ± 9.54	3.23 ± 0.37
3-epi-bufalin	963.06 ± 284.76	0.42 ± 0.14	7.74 ± 3.87	2214.18 ± 460.31	3.21 ± 0.24
Hydroxylated bufalin (RT = 1.29 min)	57.37 ± 13.41	0.58 ± 0.14	2.23 ± 0.36	144.78 ± 23.41	2.41 ± 0.07
Hydroxylated bufalin (RT = 1.63 min)	13.90 ± 4.64	0.58 ± 0.14	1.90 ± 0.37	34.45 ± 8.82	2.41 ± 0.20
Hydroxylated bufalin (RT = 2.02 min)	299.56 ± 112.89	0.50 ± 0.00	3.13 ± 1.50	643.98 ± 173.93	2.46 ± 0.17
Hydroxylated bufalin (RT = 3.42 min)	107.36 ± 44.57	0.50 ± 0.00	2.32 ± 0.71	238.03 ± 64.08	2.30 ± 0.15
Hydroxylated bufalin (RT = 3.81 min)	15.28 ± 3.86	2.67 ±1.16	12.39 ± 9.22	83.59 ± 13.61	4.06 ± 0.17
Dihydroxylated bufalin (RT = 0.84 min)	6.14 ± 1.72	0.83 ± 0.29	2.49 ± 1.05	18.80 ± 2.74	2.84 ± 0.16
Dihydroxylated bufalin (RT = 1.59 min)	14.68 ± 6.52	1.67 ± 0.58	5.81 ± 3.68	72.20 ± 22.72	3.66 ± 0.14
Dihydroxylated bufalin (RT = 2.68 min)	3.25 ± 1.68	1.17 ± 0.76	2.03 ± 0.67	11.74 ± 5.11	2.91 ± 0.11

## Supplementary Materials

Additional Supporting Information are available online.

## Abbreviations

IS	internal standard
MRM	multiple reaction monitoring
LOQ	limit of quantitation
VB	*Venenum Bufonis*
TCM	Traditional Chinese Medicine
QC	quality control
LLOQ	lower limit of quantification
RSD	relative standard deviation.
