# Supplementary material for: Simultaneous Determination of Bufalin and Its Nine Metabolites in Rat Plasma for Characterization of Metabolic Profiles and Pharmacokinetic Study by LC–MS/MS
Source: Molecules. 2019 Apr 28;24(9):1662. doi: 10.3390/molecules24091662 (PMC6539458; doi:10.3390/molecules24091662)
Supplement: Supplementary file 1 [file molecules-24-01662-s001.pdf]

Simultaneous determination of bufalin and its nine metabolites in rat plasma for characterization of metabolic profiles and pharmacokinetic study by LC-MS/MS

Wenlong Wei †, Yang Yu †, Xia Wang, Linhui Yang, Hang Zhang, Hongjian JI, Zhenwei Li, Jinjun HOU , Wanying Wu *, Dean Guo *

Affiliation

Shanghai Research Center for Modernization of Traditional Chinese Medicine, National Engineering Laboratory for TCM Standardization Technology, Shanghai Institute of Materia Medica, Chinese Academy of Science, Shanghai, 201203, China

† These two authors contributed equally to this work

Correspondence:

Prof. Wanying Wu, Shanghai Research Center for Modernization of Traditional Chinese Medicine, National Engineering Laboratory for TCM Standardization Technology, Shanghai Institute of Materia Medica, Chinese Academy of Sciences, Haike Road 501, Shanghai 201203, China.

Email: wanyingwu@simm.ac.cn Phone: +86 21 20231000 2221 Fax: +86 21 50272789

Prof. De-an Guo, Shanghai Research Center for Modernization of Traditional Chinese Medicine, National Engineering Laboratory for TCM Standardization Technology,

Shanghai Institute of Materia Medica, Chinese Academy of Sciences, Haike Road 501, Shanghai 201203, China.

Email: daguo@simm.ac.cn Phone: +86 21 20231000 2221 Fax: +86 21 50272789

Supporting Information

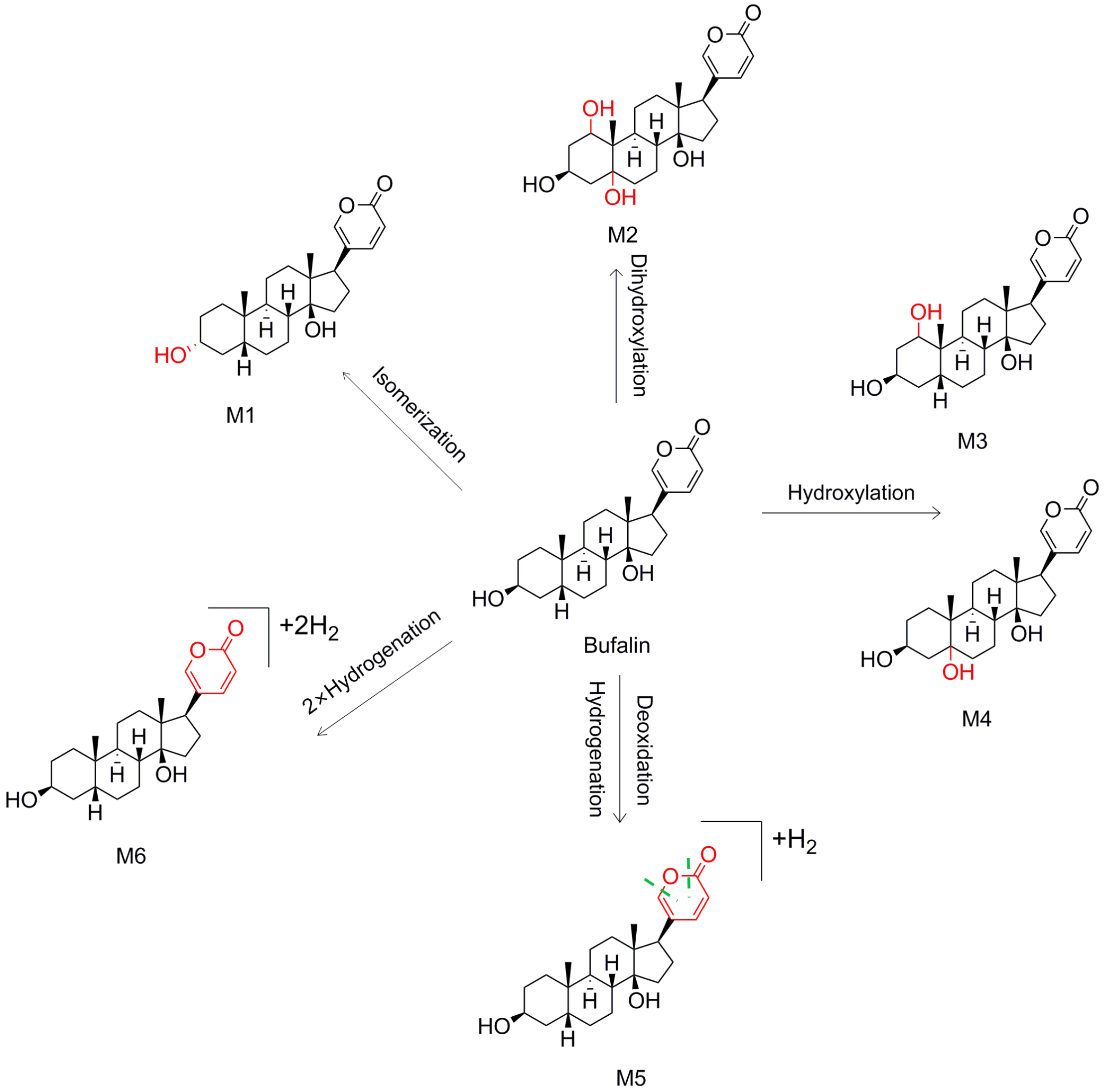

Figure S1 Proposed metabolic pathways of bufalin (M1, 3-epi-bufalin; M2, dihydroxylated bufalin; M3, 1- hydroxylated bufalin; M4, 5- hydroxylated bufalin; M5, deoxidation-hydrogenation-bufalin; M6, 2×hydrogenation-bufalin)

Table S1 The transformations included in UNIFI software

**Name**	**Deita mass (Da)**	**Formula**	**Classifier**
Decarboxylation	-43.9898	COO	phase I
Propyl ketone to acid	-40.0677	$O\text{-}C_4H_8$	phase I
Tert-butyl to alcohol	-40.0677	$O\text{-}C_4H_8$	phase I
Nitro reduction	-29.9742	H_2+O_2	phase I
Decarbonylation	-27.9949	COO	phase I
Ethyl ketone to acid	-26.052	$O\text{-}C_3H_6$	phase I
Isopropyl to alcohol	-26.052	$O\text{-}C_3H_7$	phase I
Dehydration	-18.0106	H_2O	phase I
Aromatization	-6.047	H_6	phase I
2 × Desaturation	-4.0313	H_4	phase I
Desaturation	-2.0157	H_2	phase I
Reduction	2.0157	H_2	phase I
Oxidation+desaturation	13.9793	$O\text{-}H_2$	phase I
Methylation	14.0157	CH_2	phase Π
Oxidation	15.9949	O	phase I
Hydration	18.0106	H_2O	phase I
Formylation	27.9949	CO	phase Π
2×Oxidation + desaturation	29.9742	$O_2\text{-}H_2$	phase I
2 × Oxidation	31.9898	O_2	phase I
Dihydrondiol formation	34.0055	H_2O_2	phase I
Acetylation	42.0106	C_2H_2O	phase Π
3 × Oxidation	47.9847	O_3	phase I
Proprionyl conjugation	56.0262	C_3H_4O	phase Π
Glycine conjugation	57.0251	C_2H_3NO	phase Π
Butyryl conjugation	70.0419	C_4H_6O	phase Π
Sulfation	79.9568	SO_3	phase Π
Phosphorylation	79.9663	HPO_3	phase Π
Malony	86.0004	$C_3H_2O_3$	
Cysteine conjugation	103.0092	C_3H_5NOS	phase Π
Taurine conjugation	107.0041	$C_2H_5NO_2S$	phase Π
Cysteine S-conjugation	119.0041	$C_3H_5NO_2S$	phase Π
Glutamine conjugation	129.0426	$C_5H_7O_3N$	phase Π
Pentose	132.0432	$C_5H_8O_4$	
Carnitine conjugation	144.1025	$C_7H_{14}O_2N$	phase Π
Rhamnose	146.0579	$C_6H_{10}O_4$	
Acetyl cysteine conjugation	161.0147	$C_5H_7NO_3S$	phase Π
Glucosylation	162.0528	$C_6H_{10}O_5$	phase Π
Hex	162.0528	$C_6H_{10}O_5$	
Hexose	162.0528	$C_6H_{10}O_5$	
Gysgly S adduction	176.0526	$C_5H_8N_2O_3S$	phase Π
Glucuronidation	176.0321	$C_6H_8O_6$	phase Π

Glucuronidation of carbamate	220.0219	$C_7H_8O_8$	phase Π
Glutathione conjugation	289.0732	$C_{10}H_{15}N_3O_5S$	phase Π
Glutathione S-conjugation	305.0682	$C_{10}H_{15}N_3O_6S$	phase Π

Table S2 The concentration (ng/mL) of bufalin, 3-epi-bufalin and dihydroxylatedbufalin in rats after oral administration of 10 mg/kg (n = 3)

NO. / Time (h)	Bufalin			3-epi-bufalin			Dihydroxylatedbufalin (0.84 min)			Dihydroxylatedbufalin (1.59 min)			Dihydroxylatedbufalin (2.68 min)		
	1	2	3	1	2	3	1	2	3	1	2	3	1	2	3
0.083	10.69	15.40	11.00	1065.55	519.94	267.21	0.00	0.00	0.00	0.00	0.00	0.00	0.00	0.00	0.00
0.25	10.27	19.60	14.30	1279.06	1033.45	922.14	2.93	2.13	2.21	2.03	1.50	1.17	0.84	0.73	0.75
0.50	6.69	11.80	10.70	1089.87	1073.99	1175.66	6.39	5.68	6.71	5.17	5.90	7.77	1.88	2.33	3.00
0.75	5.09	7.63	10.30	820.96	790.21	1141.46	5.65	6.96	7.17	7.80	10.28	10.37	2.18	3.65	3.27
1.00	5.50	6.65	9.95	643.93	586.15	710.87	6.58	7.50	6.64	9.70	13.45	12.42	2.36	5.04	2.92
2.00	4.63	5.50	8.24	552.04	209.80	413.29	5.52	4.14	4.44	15.00	22.03	24.27	2.66	3.92	4.02
4.00	2.61	3.07	3.14	261.50	178.05	179.88	2.24	1.65	1.60	12.57	13.05	10.87	1.63	1.55	1.31
6.00	1.30	1.66	3.20	260.14	148.32	283.71	2.57	1.66	1.67	7.58	8.92	11.55	0.64	1.45	0.88
8.00	1.60	1.19	1.68	109.33	94.94	160.87	0.97	0.75	1.87	5.82	5.77	8.05	0.11	0.59	0.97
10.00	1.22	2.01	2.34	102.44	121.96	189.74	0.32	1.49	1.70	2.15	5.73	11.36	0.40	1.09	1.78

Table S3 The concentration (ng/mL) of hydroxylatedbufalin in rats after oral administration of 10 mg/kg (n = 3)

NO. / Time (h)	Hydroxylatedbufalin (1.63 min)			Hydroxylatedbufalin (3.81 min)			Hydroxylatedbufalin (1.29 min)			Hydroxylatedbufalin (2.02 min)			Hydroxylatedbufalin (3.42 min)		
	1	2	3	1	2	3	1	2	3	1	2	3	1	2	3
0.083	7.75	3.07	0.88	0.00	0.00	0.00	32.84	11.90	4.76	190.57	77.37	40.59	57.23	27.84	20.87
0.25	11.83	12.64	7.14	1.98	1.87	1.59	51.63	43.38	34.39	253.91	260.48	243.26	79.08	88.86	78.47
0.50	14.26	17.44	14.18	5.30	5.34	6.42	65.78	67.17	62.77	265.53	352.37	376.36	87.63	136.83	129.18
0.75	12.36	18.38	16.38	7.17	8.79	8.88	62.61	67.23	73.99	206.41	333.45	385.12	83.83	130.75	133.00
1.00	11.73	16.63	11.50	11.41	11.36	10.59	59.76	61.29	54.04	161.01	267.91	255.23	78.87	115.21	106.64
2.00	8.73	6.76	8.30	17.00	16.83	22.72	37.49	26.36	38.64	119.83	94.26	158.36	50.58	39.13	60.19
4.00	2.88	3.08	2.94	16.37	12.50	18.12	13.10	10.41	10.02	53.32	48.59	53.19	16.27	16.49	16.22
6.00	2.75	2.25	2.84	12.89	11.90	14.69	12.47	10.75	13.57	51.53	45.07	89.34	17.11	15.61	24.84
8.00	1.45	1.03	2.16	11.30	6.69	13.33	5.04	5.86	9.32	22.18	27.17	51.01	6.83	8.18	17.00
10.00	0.43	1.03	2.30	7.04	6.96	17.00	3.11	8.59	10.66	15.00	35.34	57.28	4.45	10.82	18.40
